# Lysosomal and Mitochondrial Liaisons in Niemann-Pick Disease

**DOI:** 10.3389/fphys.2017.00982

**Published:** 2017-11-30

**Authors:** Sandra Torres, Elisa Balboa, Silvana Zanlungo, Carlos Enrich, Carmen Garcia-Ruiz, Jose C. Fernandez-Checa

**Affiliations:** ^1^Department of Cell Death and Proliferation, Intituto de Investigaciones Biomédicas de Barcelona, Consejo Superior de Investigaciones Científicas, Barcelona, Spain; ^2^Liver Unit and Hospital Clinc I Provincial, Centro de Investigación Biomédica en Red (CIBEREHD), Institut d'Investigacions Biomèdiques August Pi i Sunyer, Barcelona, Spain; ^3^Departamento de Gastroenterología, Facultad de Medicina, Pontificia Universidad Católica de Chile, Santiago, Chile; ^4^Departamento de Biomedicina, Unidad de Biología Celular, Centro de Investigación Biomédica CELLEX, Facultad de Medicina y Ciencias de la Salud, Institut d'Investigacions Biomèdiques August Pi i Sunyer, Universidad de Barcelona, Barcelona, Spain; ^5^Southern California Research Center for ALDP and Cirrhosis, Los Angeles, CA, United States

**Keywords:** mitochondria, lysosomes, cholesterol, sphingolipids, intracellular trafficking, lysosomal disorders, acid sphingomyelinase

## Abstract

Lysosomal storage disorders (LSD) are characterized by the accumulation of diverse lipid species in lysosomes. Niemann-Pick type A/B (NPA/B) and type C diseases Niemann-Pick type C (NPC) are progressive LSD caused by loss of function of distinct lysosomal-residing proteins, acid sphingomyelinase and NPC1, respectively. While the primary cause of these diseases differs, both share common biochemical features, including the accumulation of sphingolipids and cholesterol, predominantly in endolysosomes. Besides these alterations in lysosomal homeostasis and function due to accumulation of specific lipid species, the lysosomal functional defects can have far-reaching consequences, disrupting *intracellular* trafficking of sterols, lipids and calcium through membrane contact sites (MCS) of apposed compartments. Although MCS between endoplasmic reticulum and mitochondria have been well studied and characterized in different contexts, emerging evidence indicates that lysosomes also exhibit close proximity with mitochondria, which translates in their mutual functional regulation. Indeed, as best illustrated in NPC disease, alterations in the lysosomal-mitochondrial liaisons underlie the secondary accumulation of specific lipids, such as cholesterol in mitochondria, resulting in mitochondrial dysfunction and defective antioxidant defense, which contribute to disease progression. Thus, a better understanding of the lysosomal and mitochondrial interactions and trafficking may identify novel targets for the treatment of Niemann-Pick disease.

## Introduction

Niemann-Pick (NP) diseases encompass a group of autosomal recessive lysosomal storage disorders (LSD), characterized by the accumulation of diverse lipid species in lysosomes. While these diseases were initially considered a single entity with overlapping biochemical, pathological and clinical features, developing evidence demonstrated differential etiological causes (Patterson and Walkley, 2017; Schuchman and Desnick, 2017). Niemann-Pick type A and B (NPA and NPB) diseases are caused by deficits in the activity of acid sphingomyelinase (ASMase), an enzyme that regulates lysosomal sphingomyelin (SM) homeostasis, while Niemann-Pick type C (NPC) disease is caused by mutations in *NPC1* and *NPC2* genes, resulting in functional defects in the lysosomal proteins NPC1 and NPC2, involved in cholesterol efflux from lysosomes.

Although the primary consequence of ASMase inactivation results in the accumulation of lysosomal SM, cholesterol and other lipids types, such as bis(monoacylglycero)phosphate, glucocerebroside, GM2 and GM3 gangliosides and sphingosine also accumulate in lysosomes (Rodriguez-Lafrasse et al., 1994; Vanier, 2013; Schuchman and Desnick, 2014). Similarly, although in NPC disease cholesterol accumulation is the direct consequence of NPC1/NPC2 loss of function, SM and other sphingolipids, such as lactosylceramide, glucosylceramide, GM2 and GM3 gangliosides and sphingosine, accumulate as well (Pentchev et al., 1984; Lloyd-Evans et al., 2008; Patterson et al., 2012). Hence, these findings imply that the trafficking and metabolism of different lipid species through the endocytic pathway are severily affected in these lysosomal diseases, likely contributing to their pathogenesis. However, the molecular mechanisms and signaling pathways responsable for cell death and tissue damage in these diseases are not entirely clear.

Although both Niemann-Pick type A/B (NPA/B) and NPC diseases are caused by defects in lysosomal homeostasis and function, there are significant differences between these diseases in relation to the degree of cholesterol trafficking to mitochondria, with the consequent impact in mitochondrial dysfunction and impairment in antioxidant defense strategies. In this review, we summarize the biochemical and genetic features of both diseases, highlighting their commonalities and differences regarding lysosomal-mitochondrial cholesterol trafficking and communication as the molecular basis to understand the differential involvement of mitochondrial dysfunction in NPC disease. Further understanding the lysosomal and mitochondrial liaisons in NP diseases may thus provide the opportunity to improve and expand the current armamentarium for the treatment of these lysosomal disorders.

## NP diseases

### NPA/B disease

ASMase deficiency results primarily in the accumulation of SM in affected tissues of NPA and NPB mice and patients (Schuchman and Desnick, 2017). Patients with NPA present developmental delay, hepatosplenomegaly and progressive neurodegeneration, leading to premature death typically between 2 and 3 years of age (Schuchman and Desnick, 2014, 2017). In contrast, NPB disease presents a highly variable phenotype that is usually diagnosed in childhood by the presence of hepatosplenomegaly (Schuchman and Desnick, 2014, 2017). Most NPB patients do not exhibit neurological defects and consequently NPB patients usually live into adulthood. In the more severely affected patients, a major complication of the disease is progressive pulmonary deterioration (Schuchman and Desnick, 2014, 2017). In addition, liver damage with fibrosis and cirrhosis has been recently recognized as a relevant complication of this disease (Moles et al., 2012; Thurberg et al., 2012; McGovern et al., 2013; Lidove et al., 2015; Cassiman et al., 2016). NPA patients have a dramatic reduction in ASMase activity and typically present <5% of its physiological activity (Smith and Schuchman, 2008; Vanier, 2013; Schuchman and Desnick, 2014). In contrast, NPB patients exhibit higher ASMase residual activity, which correlates with their milder phenotype (Smith and Schuchman, 2008; Vanier, 2013; Schuchman and Desnick, 2014). *The clinical diagnosis of NPA and NPB diseases* is mainly based on the presence or absence of neurological symptons (Vanier, 2013; McGovern et al., 2017; Schuchman and Desnick, 2017). Although NPA and NPB are pan-ethnic, NPA is more frequent in individuals with Ashkenazi Jewish ancestry than in the general population, with an estimated carrier frequency close to 1:80 and a disease incidence of 1/40,000 (Schuchman and Desnick, 2014). In other populations, such as in Chile, the carrier frequency for the type B mutation A359D occurs in 90% of patients close to a 1:106 rate, predicting a disease incidence of 1/45,000 (Acuña et al., 2016a,b).

ASMase is encoded by the SMPD1 gene (sphingomyelin phosphodiesterase 1, gene ID 6609), which is located on chromosome 11 locus 11p15.4-p15.1. More than 180 pathogenic mutations in the SMPD1 gene in patients with NPA and NPB have been identified, which are concentrated in exon two (Schuchman and Desnick, 2017). Recently, the ASMase *crystal* structure has been determined in humans (Xiong et al., 2016) and mouse (Gorelik et al., 2016), which may facilitate genotype-phenotype mutation analysis (Acuña et al., 2016b; Zampieri et al., 2016).

Although tissues from mice or patients with NPA and NPB disease accumulate SM primarily in endolysosomes, cholesterol also increases as a secondary consequence (Huang and Feigenson, 1999; Radhakrishnan et al., 2000; Ridgway, 2000). While the underlying mechanism of cholesterol loading is not well understood, it is known that SM and cholesterol exhibit a high affinity for each other. Indeed, SM binds cholesterol with high affinity (Slotte, 1999; Ridgway, 2000), resulting in the sequestration and subsequent decrease in the efflux of cholesterol out of lysosomes, impairing the *esterification* of cholesterol by acyl-CoA:cholesterol acyl transferase in the ER. These events are in line with findings in macrophages from ASMase^−/−^ mice or in macrophages from wild type mice enriched with exogenous SM, which results in increased lysosomal cholesterol content because of decreased cholesterol efflux (Leventhal et al., 2001). Interestingly, it has been described that ASMase is not only active in lysosomes, but it exhibits a secretory form that acts on the plasma membrane to generate ceramide from SM hydrolysis in response to stress (Falcone et al., 2004; Charruyer et al., 2005). This event, in turn, reorganizes membrane lipid domains, called “rafts,” which trigger downstream signaling events. These observations have led to the suggestion that in addition to lysosomal dysfunction produced by SM accumulation, part of the pathogenesis of NPA and NPB is related to alterations in signaling pathways at the plasma membrane.

### NPC disease

NPC is a neurodegenerative visceral disorder with the cardinal characteristic of unesterified cholesterol accumulation in the liver, spleen and central nervous system (Patterson et al., 2012). This disease presents a broad range of symptoms, ranging from a progressive fatal neonatal disorder to a milder form in the adulthood that can evolve to chronic neurodegeneration. Although, in most cases the severity of the disease is determined by neurological deterioration, systemic signs, such as cholestatic jaundice in the neonatal period and/or hepatosplenomegaly in infancy and childhood, usually precede neurological symptoms (Patterson et al., 2012). In the most common form of NPC disease, patients exhibit progressive neurological defects. In the early period of childhood there is a delay in motor development, while in the later period of childhood and in the juvenile form symptoms manifest as gaits, falls, clumsiness, cataplexy, and school problems. In some cases, there are also psychiatric disorders such as progressive dementia (Patterson et al., 2012; Vanier, 2013).

Deterioration of liver function is a common feature of NPC disease and represents one of the most common metabolic causes of neonatal cholestasis (Patterson et al., 2012; Vanier, 2013). Currently, this disease has an estimated incidence of 1 case per 120,000 live births and is a deadly progressive disease that has so far no cure (Patterson et al., 2012).

The main cause of NPC disease is the functional inactivation of NPC1 and NPC2 proteins due to mutations in the *NPC1* and N*PC2* genes, particularly in *NPC1* gene located in chromosome 18q11, which accounts for up to 95% of cases of the disease (Carstea et al., 1993, 1997), while the remaining 5% are due to mutations in the *NPC2* gene located on chromosome 14q24 (Naureckiene et al., 2000).

NPC1 is a multi transmembrane protein located in late endosomes and lysosomes (Davies and Ioannou, 2000), while NPC2 is a relatively small protein located in the lysosomal lumen (Friedland et al., 2003). NPC1 and NPC2 work in tandem in the release of cholesterol from lysosomes. The current model of cholesterol transport in lysosomes posits that NPC2 binds cholesterol and transfers it to NPC1, which then transports cholesterol through the glycocalyx to the endosomal/lysosomal membrane to be released from the organelle by mechanisms that have not yet been determined (Kwon et al., 2009; Klein et al., 2014). The direct consequence of NPC1 deficiency is the accumulation of unesterified cholesterol in endolysosomes, which is accompanied by a secondary increase in glycosphingolipids, including SM. Consistent with the key role of NPC1 in *intracellular* cholesterol trafficking, NPC1 deficiency in mice reproduces many of the deficits seen in NPC patients, including neurological defects and ataxia by 6–7 weeks of age and severe reduction in the maximal life span to about 10–12 weeks. Experimental studies in NPC1 knockout mice have identified promising therapeutic treatment options for NPC disease (see below) and represent a valid model to examine lysosomal-mitochondrial communications that underlie the widespread defects in intracellular lipid transport.

## Mitochondria-lysosomal liaisons in NP disease

### Reciprocal functional regulation between mitochondria and lysosomes

The endo-lysosome compartment (LE/Lys) constitutes a highly dynamic membrane structure that plays a key role in the maintenance of cellular homeostasis, as well as in the digestion and recycling of cellular components and in lipid metabolism and trafficking (Luzio et al., 2007; Saftig and Klumperman, 2009; Settembre et al., 2013). Alterations in LE/Lys trafficking and lysosomal function are typical features of LSDs, such as NPA/C diseases characterized by the accumulation of specific lipid species in lysosomes, (Futerman and van Meer, 2004; Ikonen, 2008; Lloyd-Evans et al., 2008). Thus, the LE/Lys system functions like a sorting station equipped with specialized molecular devices involved in the degradation and/or recycling of a wide range of cargo. Moreover, the LE/Lys is not only involved in recycling or secretory pathways but can modulate mitochondrial function as well. For instance, Gaucher disease, a LSD caused by mutations in the *GBA1* gene that encodes for β*-glucocerebrosidase*, displays lysosomal dysfunction and defective mitochondrial turnover due to impaired mitophagy (Osellame et al., 2013), illustrating the functional relationship between lysosomes and mitochondria. In line with this notion, pharmacological and genetic models of lysosomal cholesterol accumulation have been shown to sensitize hepatocytes to acetaminophen hepatotoxicity by impairing mitophagy (see below), highlighting the relevance of lysosomes/mitochondria relationship in drug-induced liver injury (Baulies et al., 2015). In addition, mitochondria are dynamic organelles that change their distribution, structure and function in response to metabolic conditions and stress, and the molecular players involved in this process are modulated by proteins involved in intracellular trafficking (Detmer and Chan, 2007; Lapuente-Brun et al., 2013; Acín-Pérez et al., 2014). For instance, mitochondrial homeostasis is finely tuned by specific endocytic proteins, such as the GTPases dynamin-2, Drp1 (Lee et al., 2016) and EHD1, which promote mitochondrial fission (Farmer et al., 2017) or through VPS35 and the retromer complex, which regulate the turnover of the fusion protein Mfn2 (Rowland and Voeltz, 2012; Tang et al., 2015).

While these findings suggest that LE/Lys can regulate mitochondrial function, there is also evidence that mitochondrial respiration regulates the biogenesis and function of the LE/Lys compartment (Baixauli et al., 2015; Daniele and Schiaffino, 2016; Raimundo et al., 2016; Diogo et al., 2017; Elbaz-Alon, 2017). Ablation of mitochondrial oxidative phosphorylation by genetic deletion of mitochondrial transcription factor A in T cells has been shown to regulate lysosomal homeostasis and function. This event modulates T cell responses through enhanced lysosomal proliferation that *results* in defective lysosomal homeostasis, translating in decreased cathepsin B activation and ASMase inhibition, which in turn causes lysosomal SM accumulation (Baixauli et al., 2015). In line with these findings, SM accumulation has been shown to inhibit the lysosomal TRP calcium channel, impairing endolysosomal trafficking, protein degradation, and macroautophagy (Shen et al., 2012). Thus, these findings suggest that the reciprocal functional regulation of mitochondria and lysosomes may engage in a self-forward loop of potential relevance in the pathogenesis of NPA/NPC diseases, consistent with the impairment in the clearance of dysfunctional mitochondria in LSDs (Lieberman et al., 2012). Whether strategies that improve mitochondrial function (see below) have a significant impact in LSD pathology remains to be established. Therefore, dissecting the intricate communication between mitochondria and lysosomes may be critical not only for understanding essential physiological processes but also for uncovering the impact of mitochondrial dysfunction in the development of human pathologies, including LSD (Taylor and Turnbull, 2005).

### Intracellular cholesterol trafficking to mitochondria in NP disease

Despite its low content in mitochondrial membranes, the mitochondrial pool of cholesterol plays key physiological roles, including the synthesis of steroids in steroidogenic cells, bile acids in hepatocytes and the maintenance of structural and functional properties of membrane bilayers. However, as described below, NPC but not NPA disease is characterized by the accumulation of cholesterol in mitochondria in both neurons and hepatocytes by mechanisms not fully elucidated. StARD1 has emerged as one of the potential key players involved in cholesterol transport to mitochondria, as best characterized in steroidogenic cells. Indeed, StARD1 regulates the rate-limiting step of steroidogenesis, which is determined by the transfer of cholesterol from the outer mitochondrial membrane (OMM) to the inner mitochondrial membrane (IMM), where it is converted to pregnenolone by P450scc (Stocco, 2001). Mutations in the gene coding for StARD1 protein cause human congenital lipoid adrenal hyperplasia (CLAH) (Miller, 1997), highlighting the relevance of this protein in the synthesis of steroids. CLAH is characterized by defective steroidogenesis in most tissues except the placenta, which does not express StARD1 (Lin et al., 1995). StARD1, a 37 kDa protein, contains a C-terminal cholesterol binding domain and an N-terminal mitochondrial targeting sequence common to matrix proteins. The hydrophobic C-terminal binding pocket domain reversibly binds cholesterol in a 1:1 ratio (Stocco, 2001).

The trafficking of free cholesterol to OMM is mediated by several steps that involve various intracellular organelles, including lysosomes and lipid droplets (LD) and specific proteins, such as the translocator protein (TSPO) and voltage-dependent anion channel (VDAC) (Elustondo et al., 2017). Moreover, cholesterol is transferred from the OMM to the IMM by the action of StARD1 and TSPO, which associate with the mitochondrial membrane contact sites (MCS) to drive the intramitochondrial cholesterol transfer to the P450scc for metabolism and subsequent steroid formation (Rone et al., 2009). When the mitochondrial cholesterol decreases at the OMM additional free cholesterol must be moved from intracellular stores to the mitochondria. In spite of data suggesting the potential participation of TSPO in mitochondrial cholesterol trafficking, recent evidence has questioned this role. For instance, genetic models of global TSPO deletion or its specific ablation in Leydig cells showed a minimal impact in steroidogenesis and hence in mitochondrial cholesterol trafficking (Morohaku et al., 2014; Tu et al., 2014).

Interestingly, 15 genes identified by sequence homology with the StAR hydrophobic lipid-binding pocket domain of approximately 210 amino acids have been described in human and mouse (Ponting and Aravind, 1999; Iyer et al., 2001). These proteins are classified as StAR-related lipid transfer (START) domain proteins (Ponting and Aravind, 1999; Iyer et al., 2001), leading to the denomination of the original described StAR as StARD1 to denote it as the founder member of this expanding family (Alpy et al., 2001, 2009; Elustondo et al., 2017). Although, few members of the START family bind sterols (Lavigne et al., 2010; Calderon-Dominguez et al., 2014; Létourneau et al., 2015; Elustondo et al., 2017), StARD1 and StARD3 (also known as MLN64) have been implicated in cholesterol trafficking into mitochondria.

The extramitochondrial source of the cholesterol pool that reaches mitochondria is not fully understood and could originate from LD, ER, the endosomal pathway or the plasma membrane (Rone et al., 2009). In support for the plasma membrane origin of cholesterol trafficking to mitochondria mainly from LDL receptor, hormone-sensitive lipase (HSL) and the StARD1 proteins in steroidogenic tissues are thought to work together in the transport of cholesterol from plasma membrane to mitochondria (Gocze and Freeman, 1993; Freeman et al., 1998; Shen et al., 2003; Lange et al., 2009). HSL is activated upon phosphorylation mediated by cAMP-dependent protein kinase (PKA) and inhibition of HSL results in decreased steroidogenesis (Rone et al., 2009). Moreover, SNARE proteins have been shown to play a crucial role in intracelular trafficking by a cholesterol-mediated mechanism (Enrich et al., 2015; Kraemer et al., 2017). However, whether mitochondrial cholesterol targeting is regulated by SNARE-mediated trafficking remains to be established.

Another source of mitochondrial cholesterol is the ER. To reach mitochondria, cholesterol from the ER is transported by cytosolic proteins, such as the PAP7 protein, which interacts with TSPO and StARD proteins (Liu et al., 2006; Alpy et al., 2013) or through the connection of ER and mitochondria via MAMs. Recently, protein complexes that are involved in membrane contact between ER and mitochondria have been identified, but their role in lipid transport is still unclear (Elustondo et al., 2017).

Due to the relevance of lysosomal-mitochondrial liaisons in NPC, understanding the trafficking of lysosomal cholesterol to mitochondria may be essential for the pathophysiology of the disease. Interestingly, although astrocytes from NPC1 deficient mice exhibit decreased expression of StARD1 protein and mRNA levels (Chen et al., 2007), we have observed increased expression of StARD1 in liver and brain from NPC1 null mice by a poorly understood mechanism independent of ER stress (Torres et al., 2017). Besides the putative involvement of StARD1, as mentioned above, another candidate to mediate mitochondrial cholesterol trafficking in NPC is StARD3, also known as MLN64. Its N-terminus named MENTAL (MLN64 N-terminal) domain binds cholesterol (Zhang et al., 2002; Alpy and Tomasetto, 2006) and is responsible for the specific localization of the protein in the membrane of late endosomes (Alpy et al., 2001). Overexpression of StARD3 enhances steroidogenesis (Watari et al., 1997) by stimulating the mobilization of lysosomal cholesterol to the mitochondrial P450scc, whereas mutant StARD3 lacking the START domain was reported to induce cholesterol accumulation into lysosomes (Zhang et al., 2002). Interestingly, StARD3 expression increases in NPC1 cells and its overexpression in hepatocytes increases mitochondrial cholesterol and impairs mitochondrial function, reflected by decreased mitochondrial membrane potential (Balboa et al., 2017). Along with StARD3, NPC2 has been shown to contribute to the transport of endosomal cholesterol to mitochondria (Kennedy et al., 2012). In this regard, it is conceivable that the increase of mitochondrial cholesterol observed in NPC1 deficient cells could derived from the action of StARD3 and NPC2, as their expression are induced in NPC1 cells (Blom et al., 2003; Balboa et al., 2017). However, the causal role of the overexpression of these proteins in the stimulation of mitochondrial cholesterol trafficking remains to be elucidated at the molecular level. According to this potential model, the endosomal cholesterol egress mediated by StARD3 involved cholesterol binding by its MENTAL domain in the late-endosomal membranes followed by the cholesterol transfer through the cytoplasmic StART domain to a cytosolic acceptor protein or membrane (Alpy and Tomasetto, 2006).

Despite this evidence for a putative role of StARD3 in mitochondrial cholesterol trafficking, targeted mutation of the StARD3 StART domain has been shown to cause only modest alterations in cellular sterol metabolism and mice homozygous for the *Mln64* mutant allele exhibited minor perturbations in the metabolism and in the intracellular distribution of cholesterol, questioning its contribution in the intramitochondrial trafficking of cholesterol (Kishida et al., 2004). Moreover, global StARD1 deletion in mice induces lipoid adrenal hyperplasia and mice die 10 days after birth (Caron et al., 1997). These findings illustrate that other members of the StAR family cannot functionally replace StARD1, highlighting a *critical* role of this protein in steroidogenesis and hence in the trafficking of cholesterol to IMM for processing. Thus, although the understanding of the pathways of mitochondrial cholesterol trafficking and accumulation in NPC disease still remains elusive, this process is important for the progression of the disease and its further characterization may be key for the design of future therapies. Whether StARD1 in partnership with StARD3 are critical in this process remains to be fully established.

## Mitochondria-lysosomes relationship and mitochondrial quality control in NP diseases

### General overview

**Figure 1:**
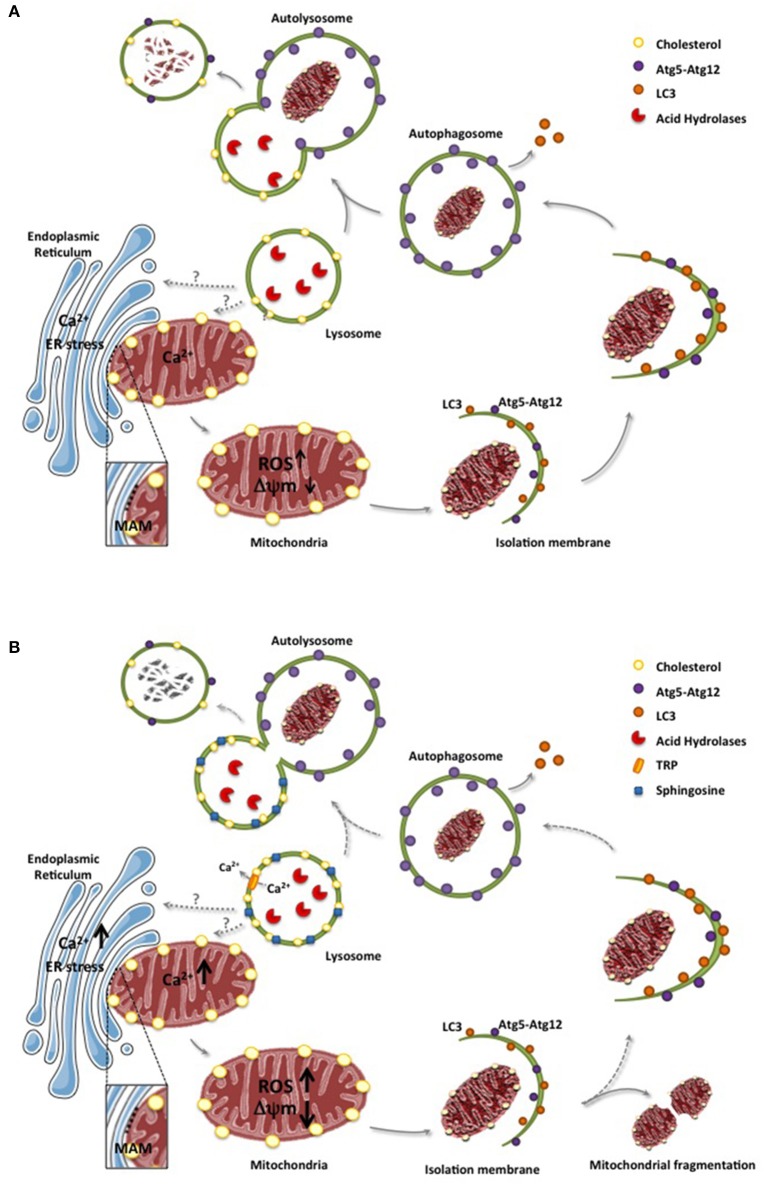
ER and lysosomes in mitochondrial degradation. **(A)**, Mitochondria and ER interaction through MAMs contributes to the uptake of calcium to mitochondria, which in physiological conditions do not cause mitochondrial dysfunction. However, in conditions of ER stress, enhanced flux of calcium from ER to mitochondria can cause oxidative stress, increased generation of reactive oxygen species (ROS) and mitochondrial depolarization. As a mechanism to ensure the elimination dysfunctional mitochondria, altered mitochondria is engulfed in sequential structures originating with the isolation membrane, which evolve to form autophagosomes. These structures encapsulating dysfunctional mitochondria are fused with lysosomes to form autolysosomes where mitocondria contained in the autolysosomes are degraded by acid hydrolases. **(B)**, In LSD, such as NPA or NPC, lipid species accumulate in lysosomes, including sphingosine and cholesterol, which not only disrupt lysosomal calcium homeostasis through calcium channels (e.g., TRP) but also impair the fusion of lysosomes with autophagosomes leading to defective mitochondrial degradation. Moreover, defective engulfment of dysfunctional mitochondria may occur in earlier steps, which may favor the fragmentation of mitochondria. Of note, while the physical association between ER and mitochondria through MAMs are relatively well defined, the physical apposition between lysosomes and mitochondria is poorly understood and characterized. Dashed lines reflect defective steps in LSD leading to impaired autolysosome formation and defective digestion of dysfunctional mitochondria.

Mitochondria are double-membrane organelles that are essential for energy supply, metabolism, production of reactive oxygen species and apoptosis signaling (Hatefi, 1985). Mitochondrial function is particularly important in tissues with high energy demand, such as the brain (Chan, 2006), which reflects the dependence of neurons on oxidative phosphorylation for energy supply (Almeida et al., 2001). Given the central role of mitochondria in cellular homeostasis, mitochondrial dysfunction has been linked to neurodegenerative diseases, including NPC (Johri and Beal, 2012; Plotegher and Duchen, 2017). Moreover, limited degradation of dysfunctional mitochondria through different mechanisms may contribute to LSD pathognesis. The degradation of damaged mitochondria can occur either by a selective process called mitophagy or non-specifically via macroautophagy (Youle and Narendra, 2011). The degradation of mitochondria is not only dependent on lysosomal function, but also on the formation of autophagosomes and the subsequent fusion of autophagosomes with lysosomes to generate autolysosomes (Figure 1A), a step that can be influenced by the lipid composition of lysosomes.

Besides Parkin and PINK1, which play a key role in mitophagy (McLelland et al., 2016), recent findings have shown that the targeting of mitochondrial components to lysosomes involves the formation of novel structures called mitochondrial-derived vesicles (MDVs) that incorporate selective mitochondrial-derived cargo for degradation in lysosomes (Soubannier et al., 2012; Schrader et al., 2015). However, unlike classical mitophagy, the delivery of MDV-containing oxidized mitochondrial components to lysosomes does not require mitochondrial depolarization and is independent of ATG5 and LC3. Thus, this alternative pathway is distinct from mitophagy and can be regarded as a novel vesicle transport route between the mitochondria and lysosomes, emerging as a complimentary mechanism for the quality control of mitochondria of potential relevance in NP disease (Soubannier et al., 2012).

### Mitochondrial quality control in NP disease

A considerable body of evidence suggests that impaired autophagy contributes to lysosomal lipid storage in LSDs through the accumulation of ubiquitinated proteins and dysfunctional organelles, including mitochondria (Platt et al., 2012; Osellame and Duchen, 2014). Although the formation of autophagosomes is enhanced in NPC neurons, the fusion of autophagosome with lysosomes is incomplete, translating in the inefficient degradation of autophagosome-containing mitochondria, which leads to the accumulation of defective mitochondria (Figure 1B; Elrick et al., 2012; Ordonez, 2012). As neurons are more sensitive to mitochondrial failure than other cells, defects in the mitochondria quality control in neurons may explain the selective neuronal loss observed in NPC disease. In line with this concept, it has been shown that defective mitophagy and increased mitochondrial fragmentation due to abnormal autophagy activation are more severe in neurons from NPC1^−/−^ mice than in NPC fibroblasts (Ordonez, 2012). Moreover, decreased NPC1 function in neurons generated from human embryonic stem cells results in impaired mitochondrial clearance but enhanced mitochondrial fragmentation, phenotypes that can be rescued by inhibition of autophagy with 3-methyladenine or cholesterol extraction with cyclodextrin. These findings suggest that mitochondrial dysfunction in NPC disease may be the consequence not only of defective autophagy induction but also of increased mitochondrial fragmentation mediated by impaired autophagosome formation (Ordonez et al., 2012).

Another factor that can contribute to impaired autophagy and defective clearance of dysfunctional mitochondria is the accumulation of sphingosine in lysosomes, which has been shown to disrupt calcium homeostasis (Lloyd-Evans et al., 2008). While the mechanism of lysosomal sphingosine accumulation in NPC is not completely understood, it has been shown that alteration in the VGEF signaling pathway impairs sphingosine kinase activity, which results in enhanced sphingosine levels and loss of Purkinje neurons via inhibition of autophagosome-lysosome fusion, thus suggesting a link between sphingosine and impaired autophagy in NPC (Lee et al., 2014). Furthermore, NPC2 deficiency impairs autophagy-lysosomal activity, which negatively impacts mitochondrial function in adipocytes (Guo et al., 2016). Interestingly, stimulation of adenosine A2A receptors has been shown to rescue intracellular cholesterol accumulation and mitochondrial abnormalities in cell models of NPC (Ferrante et al., 2016), linking A2A agonism with the improvement of mitochondrial function and pathological phenotype of fibroblasts from NPC patients (Visentin et al., 2013). Therefore, the accumulation of dysfunctional mitochondria can account for the onset of oxidative stress in NPC disease described both in NPC1 knockout mice and NPC patients (Vázquez et al., 2012). Moreover, in addition to impaired mitophagy, defects in mitochondrial motility in neurons and distribution into axons to meet metabolic demands can contribute to the pathogenesis of neurological disorders, including NPC disease (Sheng and Cai, 2012; Woś et al., 2016). However, whether these alterations are relevant for the functional maintenance of Purkinje neurons, whose loss accounts for the characteristic ataxia in NPC disease remains to be established.

In parallel with these findings in NPC, there is also evidence of autophagy defects in NPA/B diseases (Fucho et al., 2014; Baulies et al., 2015; Canonico et al., 2016). For instance, hepatocytes from ASMase^−/−^ mice exhibit impaired autophagy flux determined by the combination of rapamycin with or without chloroquine, an effect that was accompanied by increased LC3BII and p62 levels. In addition, ASMase^−/−^ hepatocytes displayed impaired fusion of autophagosome-containing mitochondria with lysosomes in response to acetaminophen, which translated in increased susceptibility to acetaminophen-mediated liver injury by sustaining mitochondrial damage (Baulies et al., 2015). Although ASMase deficiency results primarily in increased lysosomal SM content, it also causes lysosomal cholesterol accumulation, which accounts for the sensitization to acetaminophen-induced hepatocellular injury. In line with these findings, U18666A, an amphiphilic amino-steroid that induces lysosomal cholesterol accumulation sensitized primary hepatocytes to acetaminophen-induced cell death. Treatment with oxysterol 25-hydroxycholesterol, a ligand for liver X receptors (LXR) that suppresses sterol synthesis, decreases cholesterol accumulation and protects ASMase^−/−^ mice from acetaminophen-mediated liver injury. Moreover, human B lymphocytes from patients with NPB disease exhibit alterations in the rate of autophagic vacuole accumulation, mitochondrial fragmentation and mitophagy induction, indicating impaired clearance of damaged mitochondria (Canonico et al., 2016). Thus, taken together these findings indicate that defective clearance of dysfunctional mitochondria due to impaired lysosomal function or defective fusion of lysosomes with autophagosomes is a common feature of both NPA/B and NPC diseases.

## Mitochondrial cholesterol accumulation: a differential feature between NPA/B and NPC diseases

**Figure 2:**
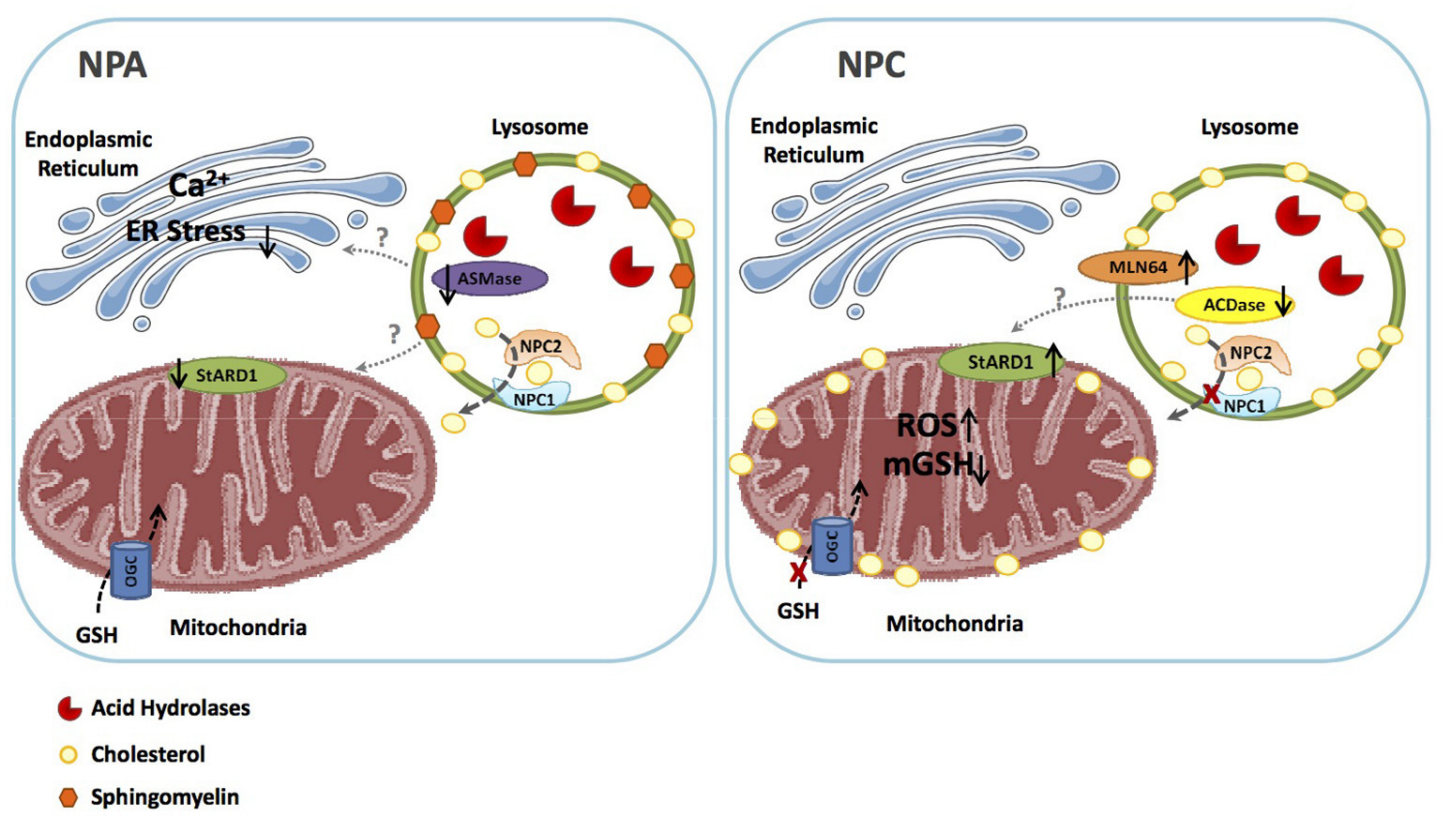
Similarities and differences between NPA and NPC. NPA disease due to ASMase deficiency is characterized by the primary accumulation of shingomyelin in lysosomes, which secondarily lead to the sequestration of cholesterol in these organelles. As ASMase triggers ER stress, the ablation of ASMase impairs ER stress signaling, and consequently the expression of StARD1. Thus, in NPA the bulk of cholesterol accumulation is restricted to lysosomes but not mitochondria, which allows the transport of GSH into the mitochondrial matrix via the 2-oxoglutarate carrier (OGC). In NPC, however, although primarily accumulates in lysosomes, mitochondrial cholesterol transporting polypeptides, such as MLN64 contribute to mitochondrial cholesterol loading. Moreover, besides MLN64, StARD1 is also induced in NPC cells by a poorly understood mechanism independent of ER stress, with the potential involvement of the downregulaton of acid ceramidase, which has been shown to repress StARD1 expression. The subsequent mitochondrial cholesterol accumulation then impairs the transport of GSH into mitochondria, resulting in mitochondrial GSH depletion and subsequent oxidative stress. These findings constitue the basis for the potential beneficial effects of mGSH replenishment by GSH ethyl ester in NPC.

In contrast to defective autophagy, which is common to NPA and NPC, intracellular cholesterol trafficking and accumulation in mitochondria is a differential feature between NPA/B and NPC diseases (Figure 2). Although the primary feature of NPA is the accumulation of SM due to the deficiency of ASMase, cholesterol levels also increase through the regulation of SREBP2. However, the bulk of the intracellular cholesterol accumulation in NPA is sequestered in lysosomes as shown in hepatocytes from ASMase null mice stained with filipin and lysotracker (Fucho et al., 2014). In contrast to this event, there is growing evidence of increased mitochondrial cholesterol loading in both hepatocytes and neurons from Npc1^−/−^ mice (Figure 2), and this process mediates, in part, the mitochondrial dysfunction reported in NPC disease (Yu et al., 2005; Marí et al., 2006; Charman et al., 2010; Balboa et al., 2017; Torres et al., 2017). Moreover, while alcohol induced the trafficking and accumulation of cholesterol in hepatic mitochondria, this event was defective in hepatocytes deficient in ASMase, which paralleled the lack of induction of StARD1 in ASMase null mice in response to alcohol feeding (Fernandez et al., 2013). The mechanism underlying the differential increase in mitochondrial cholesterol and expression of StARD1 between NPA and NPC is not currently understood (Torres et al., 2016). As acid ceramidase (ACDase) has been shown to repress steroidogenic factor-1-dependent expression of StARD1 (Lucki et al., 2012), it remains to be established whether decreased ACDase in NPC disease contributes to the upregulation of StARD1 and subsequent mitochondrial cholesterol loading (Figure 2), which is currently under investigation. Moreover, as mentioned above, NPC2 has been shown to contribute to the transport of endosomal cholesterol to mitochondria independently of NPC1 (Kennedy et al., 2012). As the loss of function of NPC2 accounts for a minor fraction of NPC cases, it is conceivable that NPC2 may play a role in the trafficking of endosomal cholesterol to mitochondria in most NPC patients (Kennedy et al., 2012).

Increased mitochondrial cholesterol content in NPC cells can lead to important functional consequences, such as decreased mitochondrial membrane fluidity (Colell et al., 2003), reduced ATP generation (Echegoyen et al., 1993; Yu et al., 2005), and decreased mitochondrial GSH (mGSH) import (Marí et al., 2006; Garcia-Ruiz et al., 2009). In addition, mitochondrial membrane potential and the activity of ATP synthase are markedly decreased in NPC1 mouse brains and neurons (Yu et al., 2005; Balboa et al., 2017; Torres et al., 2017) and the inhibition of ATP synthase is reversible upon mitochondrial cholesterol extraction by cyclodextrin (Yu et al., 2005). In line with these findings, increased mitochondrial cholesterol loading triggers metabolic adaptations in NPC models, characterized mainly by enhanced glycolysis (Kennedy et al., 2014). The depletion of mGSH levels has a severe impact in NPC disease, as inferred by its replenishment with GSH ethyl ester (GSH-EE), which restored the mGSH pool in liver and brain of Npc1^−/−^ mice and in fibroblasts from NPC patients, leading to increased median survival and maximal life span of Npc1^−/−^ mice, protection against oxidative stress and oxidant-induced cell death and restoration of calbindin levels in cerebellar Purkinje cells, which improved locomotor impairment in Npc1^−/−^ mice. In addition, high-resolution respirometry analyses showed that GSH-EE treatment improved oxidative phosphorylation, coupled respiration and maximal electron transfer in cerebellum of Npc1^−/−^ mice (Torres et al., 2017). In contrast, the antioxidant and cytosolic GSH precursor N-acetylcysteine (NAC), which increased total GSH levels in liver and brain homogenates from Npc1^−/−^ mice (Fu et al., 2013), failed to restore the mGSH pool (Torres et al., 2017). These results are in agreement with the notion that increased cholesterol content in NPC mitochondria disrupts membrane dynamics and impairs the transport of GSH from the cytosol into mitochondria (Marí et al., 2009), contributing to the activation of apoptotic pathways seen in NPC mutant cells (Klein et al., 2011). In line with the role of oxidative stress, it has been shown that vitamin E supplementation in the diet delayed weight loss, improved coordination and locomotor function and increased the survival of Npc1^−/−^ mice (Marín et al., 2014). Moreover, vitamin E supplementation preserved Purkinje neurons and reduced levels of astrogliosis, nitrotyrosine and apoptotic signaling mediated by the c-Abl/p73 pathway (Marín et al., 2014). Part of this beneficial effect could be related to increased levels of α-tocopherol in mitochondria, which has been shown to quench ROS production, especially in hepatic mitochondria (Chow et al., 1999), thus preventing GSH depletion (Stojkovski et al., 2013).

In addition to these events, mitochondrial cholesterol loading may impair mitochondrial dynamics reflected in the balance between fusion and fission events. Disruption of appropriate mitochondrial fluidity following cholesterol accumulation can prevent the fusion of mitochondria with adjacent healthy mitochondria (Baker et al., 2014), leading to increased predominance of fragmented mitochondria. In line with these events, we have recently reported that mitochondria from NPC cells exhibited a more rounded and smaller morphology compared to wild type cells, paralleling the decrease in mitochondrial membrane potential and increased mitochondrial superoxide production (Balboa et al., 2017). Finally, disruption of lysosomal-mitochondrial interplay can also have an impact in the Ca^2+^ buffering capacity of mitochondria (Kiselyov and Muallem, 2008) in LSDs, in line with the effect of suppressing lysosomal function and autophagy in mitochondrial Ca^2+^ homeostasis (Jennings et al., 2006). As mentioned above, it has been recently shown that NPC disease is characterized by the disruption of lysosomal Ca^2+^ homeostasis preceded by the accumulation of sphingosine that impairs the endocytic pathway (Lloyd-Evans et al., 2008). Whether this event is linked to altered mitochondrial Ca^2+^ content and function remains to be established. In addition, further work is needed to demonstrate a causal role for the disruption of Ca^2+^ homeostasis in the death of the Purkinje neurons in NPC disease, as shown in other related diseases (Girard et al., 2012; Kasumu and Bezprozvanny, 2012).

## Therapeutic approaches and concluding remarks

Although the genetic causes of NPA and NPC disease are well understood, the consequences at the level of disruption of intracelular trafficking and interorganelle communication is still incomplete, thus limiting the availability of effective therapy. Enzyme replacement therapy for ASMase and NPC1 deficiency is expected to successfully treat peripheral non-neuronal symptoms of both diseases (Schuchman and Desnick, 2017). However, as this approach may be inefficient to replenish the defective enzymes in the brain due to the blood brain barrier, the expectations for the correction of the neurological symptoms may be disapointing. Therefore, there is the urgent need for alternative possibilites to target both diseases exploiting the fact that several intracelular compartments are involved in the pathogenesis of NPA and NPC diseases (Saffari et al., 2017). In the case of NPC, in which mitochondrial dysfunction and cholesterol accumulation have been well established, strategies targeting mitochondrial cholesterol accumulation and/or downstream consequences may be of potential relevance. In this scenario, recent data indicated that the replenishment of mGSH levels by permeable GSH prodrugs have shown promising results in fibroblasts from NPC patients, correcting the mitochondrial dysfunction and ameliorating oxidative stress, while extending the survival of NPC1 null mice (Torres et al., 2017). Quite intriguingly, the efficacy of mGSH replenishment by GSH-EE was not potentiated by parallel treatment with cholesterol extraction with cyclodextrin, suggesting that both processes are related to each other. This outcome raises the possibility that the efficacy of mGSH restoration by GSH-EE could be explored in combination with other therapies targeting differential pathways. For instance, since curcumin has been shown promising results by restoring intracellular sphingosine balance and cytosolic calcium homeostasis (Lloyd-Evans et al., 2008), it may be worth investigating the combination of curcumin with GSH-EE in NPC disease. Moreover, recent evidence has shown a beneficial role for histone deacetylase (HDAC) inhibitors (i.e., vorinostat or valproic acid) as a novel line of therapy for NPC disease, resulting in the reduction of cholesterol accumulation, improvement of neurodegenerative symptoms and visceral alterations, which culminate in the extension of mouse life span (Kim et al., 2007; Pipalia et al., 2011; Alam et al., 2016; Contreras et al., 2016; Munkacsi et al., 2017). As HDAC can regulate mitochondrial function, it may be worth to test whether combined treatment with HDAC inhibitors and GSHEE would exhibit greater therapeutic benefit in NPC1 mutant mice with respect to either agent alone, supportingh this approach as a promising combination therapy for NPC.

## Author contributions

ST, EB, SZ, CE, CG-R, and JF-C discussed findings, analyzed literature and wrote the manuscript. ST, CG-R, and JF-C designed the schematic Figures.

### Conflict of interest statement

The authors declare that the research was conducted in the absence of any commercial or financial relationships that could be construed as a potential conflict of interest.

## Abbreviations

ACDase: acid ceramidase
ASMase: acid sphingomyelinase
CLAH: congenital lipoid adrenal hyperplasia
ER: endoplasmic reticulum
GSH-EE: GSH ethyl ester
GM2: ganglioside GM2
GM3: ganglioside GM3
HSL: hormone-sensitive lipase
IMM: inner mitochondrial membrane
LD: lipid droplets
LSDs: lysosomal storage disorders
LE/Lys: late endosomes/lysosomes
mGSH: mitochondrial GSH
MAM: mitochondrial associated ER membranes
MCS: membrane contact sites
NAC: N-acetylcysteine
NP: Niemann-Pick diseases
NPA: Niemann-Pick type A disease
NPB: Niemann-Pick type B disease
NPC: Niemann-Pick type C disease
NPC1: Niemann-Pick type C1 protein
NPC2: Niemann-Pick type C2 protein
OMM: outer mitochondrial membrane
SM: sphingomyelin
SMPD1: sphingomyelin phosphodiesterase 1
SREBP2: sterol regulatory element protein 2
StARD1: steroidogenic acute regulatory protein 1
StARD3: steroidogenic acute regulatory protein 1
START: StAR-related lipid transfer domain
VDAC: voltage dependent anion channel
TSPO: translocator protein.
